# Joint–brain communication in rheumatoid arthritis: glial mechanisms, pain phenotypes, and translational evidence

**DOI:** 10.3389/fimmu.2026.1889608

**Published:** 2026-09-29

**Authors:** Dong Guo, Ke Xu, Lin Xiao, Hui Li, Zheng Li, Ying Pan, Huan Zhang

**Affiliations:** Department of Joint Surgery, Honghui Hospital, Xi’an Jiaotong University, Xi’an, China

**Keywords:** central sensitization, glial cells, joint–brain axis, neuroinflammation, rheumatoid arthritis

## Abstract

Rheumatoid arthritis (RA) is a systemic autoimmune disease in which persistent pain, fatigue, cognitive impairment, depression, and anxiety may involve altered central nervous system (CNS) processing. This narrative review synthesizes preclinical and clinical evidence concerning proposed glial cell-mediated joint–brain communication in RA. Joint-derived neural inputs and systemic inflammatory signals may communicate with the CNS through humoral, cellular, neural, neuroendocrine, and neurovascular pathways. Preclinical studies implicate microglia and astrocytes in neuroinflammatory responses, spinal central sensitization, impaired hippocampal plasticity, and neurovascular alterations. Human studies associate systemic inflammation with altered brain connectivity and CNS-related symptoms. Disease-modifying antirheumatic treatment, particularly tumor necrosis factor inhibition, has been associated with improvements in selected CNS-related outcomes. Adjunctive symptom management includes phenotype-guided analgesic approaches, rehabilitation, and psychological and sleep-focused interventions. Translational research explores glial modulators, pathway-specific interventions, vagal neuromodulation, neuroimaging, cerebrospinal fluid markers, and multidimensional patient stratification. The proposed joint–brain framework highlights the value of assessing inflammation alongside pain phenotype, cognition, mood, fatigue, sleep, and function. Future human studies should evaluate whether mechanistic and biomarker findings can improve individualized treatment selection and clinical outcomes.

## 1 Introduction

### 1.1 Rheumatoid arthritis as a systemic disease with CNS-related clinical burden

Rheumatoid arthritis (RA) is a chronic autoimmune disease affecting approximately 0.5–1% of the global population, traditionally defined by progressive joint destruction and systemic inflammation (1). However, patients with RA frequently experience persistent pain, cognitive symptoms, depression, and anxiety; these outcomes are multifactorial and have been associated with systemic inflammation, altered CNS processing, comorbidities, and psychosocial factors (2–4).

Chronic pain is among the most disabling RA-associated manifestations and may persist even when peripheral inflammation is well controlled (5). Cognitive symptoms can affect attention, memory, and executive function, while depression and anxiety further reduce quality of life and functional recovery (2, 4, 6).

A key clinical challenge is the disconnect between peripheral disease control and persistent CNS-related symptoms. Neuroimaging studies in patients with RA have reported associations between systemic inflammatory activity and alterations in brain regions involved in pain, cognition, and emotion, while small interventional studies suggest that some imaging abnormalities may change after cytokine blockade (7, 8). These observations support broader clinical assessment of persistent symptoms in RA (9, 10). Throughout this review, “CNS-related” is used descriptively for symptoms or findings involving central processing.

### 1.2 The proposed joint–brain axis and potential glial contributions

The proposed joint–brain axis encompasses neural signals originating from inflamed joints together with circulating and neuroendocrine signals associated with systemic RA inflammation; their relative contributions remain incompletely resolved. Candidate communication routes include cytokine sensing at neurovascular and circumventricular interfaces, immune-cell trafficking, joint-afferent and autonomic pathways, and neuroendocrine regulation. Evidence for this framework derives from experimental arthritis models, human neuroimaging studies, and a randomized clinical neuromodulation trial, although the strength and directness of evidence differ among these sources (11–13).

Within the CNS, microglia and astrocytes are candidate mediators of responses to peripheral inflammatory and neural signals, with proposed effects on cytokine signaling, spinal nociceptive processing, and synaptic plasticity. Most cell-specific and pathway-level evidence derives from experimental arthritis and related inflammatory pain models; direct demonstration of these glial responses and their clinical consequences in patients with RA remains limited (14, 15). The pathways and predominant levels of supporting evidence for bidirectional joint–brain communication are summarized in **Figure 1**.

**Figure 1:**
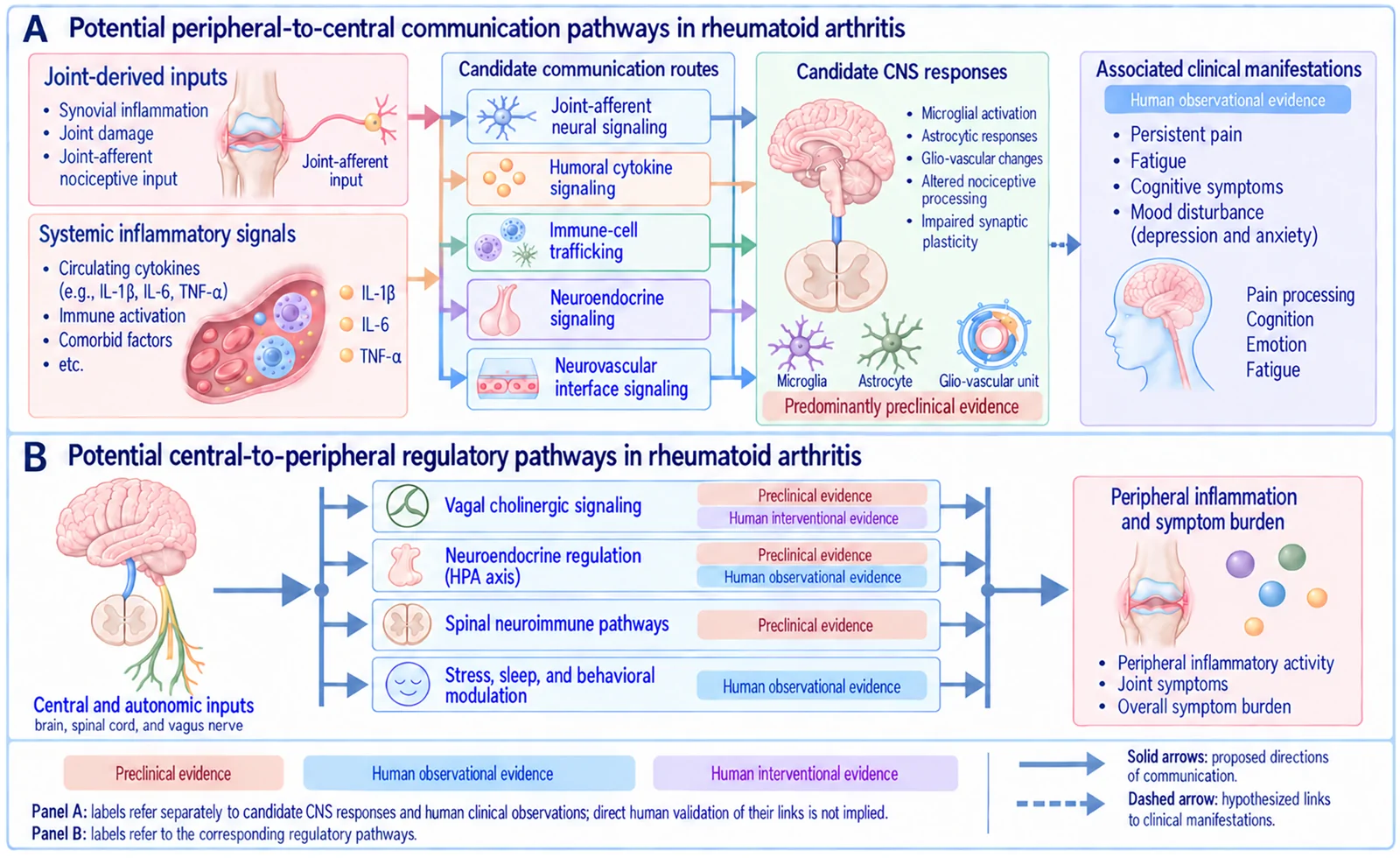
Proposed bidirectional joint–brain communication pathways in rheumatoid arthritis and the predominant levels of supporting evidence. **(A)** Peripheral-to-central communication. Joint-derived inputs and systemic inflammatory signals may communicate with the central nervous system through joint-afferent neural signaling, humoral cytokine signaling, immune-cell trafficking, neuroendocrine signaling, and neurovascular interface signaling. Candidate CNS responses include microglial activation, astrocytic responses, glio-vascular changes, altered nociceptive processing, and impaired synaptic plasticity, which may contribute to persistent pain, fatigue, cognitive symptoms, and mood disturbance. Evidence labels refer separately to the predominantly preclinical findings concerning candidate CNS responses and human observational findings concerning clinical manifestations; they do not imply direct human validation of the links between them. **(B)** Central-to-peripheral regulation. Central and autonomic inputs may influence peripheral inflammatory activity and symptom burden through vagal cholinergic signaling, neuroendocrine regulation, spinal neuroimmune pathways, and stress-, sleep-, and behavior-related modulation. Evidence labels refer to the corresponding regulatory pathways; spinal neuroimmune modulation of peripheral arthritis is supported by preclinical evidence. Solid arrows indicate proposed directions of communication, whereas the dashed arrow indicates hypothesized links between candidate CNS responses and clinical manifestations. CNS, central nervous system; HPA, hypothalamic–pituitary–adrenal; IL, interleukin; TNF, tumor necrosis factor.

### 1.3 Scope of this review

This article is a narrative review rather than a systematic review or meta-analysis and is intended for a broad clinical and translational readership across rheumatology, pain medicine, neuroscience, and immunology. It integrates preclinical and clinical evidence on proposed links between RA and CNS-related features, focusing on candidate joint–brain communication pathways and possible roles of microglia and astrocytes. For clarity, evidence is classified as preclinical (experimental arthritis models, related non-RA models, or cell-based studies), human observational (cohort, neuroimaging, cerebrospinal fluid, or patient-reported studies), or human interventional (treatment or neuromodulation studies).

Relevant studies were identified through searches of PubMed, Web of Science, Scopus, and Google Scholar up to the time of manuscript preparation. Search terms included combinations of “rheumatoid arthritis”, “central nervous system”, “joint–brain axis”, “neuroinflammation”, “microglia”, “astrocytes”, “central sensitization”, “pain”, “depression”, “cognitive dysfunction”, “cytokines”, and “vagus nerve stimulation”. Reference lists of key reviews and original studies were also screened.

Emphasis is placed on translational implications. By comparing experimental arthritis models with clinical evidence from neuroimaging, cerebrospinal fluid (CSF) analysis, neuromodulation, and pharmacological studies, this review evaluates candidate glial pathways identified mainly in preclinical research. Their relevance to human RA and treatment selection remains to be established. It also discusses current barriers, including CNS heterogeneity, inconsistent responses to glial modulators, and insufficient patient stratification.

## 2 CNS-related clinical features and supporting evidence in RA

### 2.1 Spectrum of CNS-related symptoms

Chronic pain. Chronic pain is among the most prevalent and disabling symptoms in RA and may persist despite adequate control of peripheral inflammation. In a prospective multicenter cohort, 22.6% of RA patients developed severe persistent pain despite meaningful improvement in disease activity, with pain catastrophizing and tender joint count, rather than inflammatory markers, predicting persistence (16). Similarly, 38.4% of patients in a biologics-treated French cohort continued to report moderate-to-severe pain, even though many were in clinical remission (17). Large trajectory analyses further showed that persistent pain subgroups remained common, including among patients with normalized inflammatory markers (18). These cohort data suggest that peripheral inflammation alone does not fully explain persistent pain.

Cognitive impairment and dementia risk. Cognitive dysfunction is increasingly recognized in RA, affecting attention, memory, executive function, and verbal fluency. In older patients with RA, cognitive impairment defined using the Montreal Cognitive Assessment (MoCA) was reported in 72% and was associated with higher disease activity (19). Meta-analytic evidence confirms moderate impairments across multiple cognitive domains in immune-mediated inflammatory diseases, with RA showing a burden comparable to systemic lupus erythematosus (20). Cognitive deficits may also appear early, with 20% of patients within three years of RA diagnosis meeting criteria for cognitive impairment (21). Some observational and Mendelian randomization studies have reported an association between RA and Alzheimer’s disease risk (22, 23); however, epidemiological findings remain heterogeneous, and the underlying relationship requires further clarification (24).

Depression, anxiety, and fatigue. Depression and anxiety are common RA comorbidities and are associated with greater pain, poorer function, and lower quality of life (4, 6, 25). In the prospective multicenter observational VADERA II study of patients with RA, 55.4% had at least mild depressive symptoms, yet only 11.7% received antidepressant therapy (6). Depression severity was associated with higher disease activity, fatigue, and lower circulating brain-derived neurotrophic factor (BDNF) levels (25). Fatigue is similarly frequent and persistent: early RA cohorts report fatigue in 44% and severe fatigue in 19%, with limited improvement over time (26). Importantly, fatigue correlates weakly with inflammatory markers but strongly with pain, mental health, and function, supporting a multifactorial interpretation involving inflammatory, psychological, and potentially central mechanisms (27).

Together, these studies document substantial pain, cognitive, affective, and fatigue-related burden in RA. These multifactorial manifestations likely reflect interactions among inflammatory, neural, psychological, and functional factors.

### 2.2 Neuroimaging evidence and its limitations

Structural neuroimaging studies in patients with RA have reported alterations in limbic and white matter regions. In a translational study that also included experimental arthritis, pain and functional disability in patients with RA were associated with hippocampal volume differences (28). UK Biobank analyses further identified smaller amygdala volume in RA patients and increased white matter hyperintensity burden compared with matched controls (29, 30). Reduced white matter integrity has been reported to statistically mediate associations between RA and depression, sleep disturbance, and dementia-related outcomes (31, 32). Grey matter loss in the inferior parietal lobule has additionally been associated with peripheral inflammation and clinical symptoms (12).

Functional magnetic resonance imaging (MRI) studies show abnormal connectivity in networks related to pain, attention, and affective regulation. In a multimodal MRI study of patients with RA, systemic inflammatory activity was associated with altered connectivity involving the inferior parietal lobule, medial prefrontal cortex, and distributed brain networks, and these connectivity patterns were associated with fatigue, pain, and cognitive symptoms (12). RA patients with fibromyalgianess show increased default mode network–insula connectivity, supporting the concept that RA may include nociplastic or centrally amplified pain components (33). In patients with RA and concomitant fibromyalgia, erythrocyte sedimentation rate (ESR) was associated with insula–parietal–cingulate connectivity (34). Fatigue is also linked to increased coupling between the dorsal attention network and medial prefrontal regions, potentially reflecting inefficient network switching (35, 36).

Anti-inflammatory treatment may be associated with changes in RA-related brain abnormalities. Human neuroimaging studies have reported rapid changes in pain-related brain activation and subjective well-being after tumor necrosis factor (TNF) inhibition, in some cases preceding measurable improvement in joint swelling (37, 38). Longitudinal and dynamic connectivity studies have also reported reductions in inflammation-associated network patterns after effective therapy (12, 39). Treatment-associated imaging changes may reflect reduced peripheral inflammation and altered afferent input, as well as changes in central processing. Some CNS-related abnormalities persist despite peripheral disease control (7). In experimental arthritis, hippocampal TNF can remain elevated after arthritis resolution and is associated with persistent pain and depression-like behavior (14).

### 2.3 Clinical pain phenotypes in RA: nociceptive, neuropathic-like, and nociplastic features

Pain in RA may include overlapping nociceptive, neuropathic-like, and nociplastic features (40, 41). Nociception is the neural process of encoding stimuli that damage or threaten to damage tissue; it is distinct from the subjective experience of pain. Nociceptive pain results from activation of nociceptors in response to actual or threatened damage to non-neural tissue. Neuropathic pain arises from a lesion or disease of the somatosensory nervous system, whereas “neuropathic-like” describes suggestive symptoms without a confirmed causative lesion or disease. Nociplastic pain arises from altered nociceptive processing and is not fully explained by nociceptive or neuropathic mechanisms (5, 40, 41). Thus, “nociceptive processing” refers to neural processing rather than a specific pain phenotype, and its use in discussing nociplastic pain does not imply classification as nociceptive pain. These pain mechanisms may coexist in patients with RA (40, 41). Active synovitis and joint damage generate nociceptive input and may produce peripheral sensitization, defined as increased responsiveness and reduced thresholds of peripheral nociceptors (40). Central sensitization is a neurophysiological mechanism characterized by increased responsiveness of nociceptive neurons within the CNS (40). Although related, nociplastic pain and central sensitization are not interchangeable concepts (40, 41). Clinically, central sensitization can only be inferred indirectly from findings such as widespread hyperalgesia, allodynia, temporal summation, or impaired conditioned pain modulation; these findings do not provide cell-specific evidence of CNS pathology (40, 42). A systematic review of 27 quantitative sensory testing studies reported reduced pressure pain thresholds in most controlled comparisons between patients with RA and healthy controls (42), while inflammatory indices and quantitative sensory testing measures explained only part of the variance in pain severity (43, 44). Experimental arthritis studies further implicate spinal glial activation and signaling involving the N-methyl-D-aspartate receptor subunit 2B (NR2B) and extracellular signal-regulated kinase 2 (ERK2), but direct validation of these cellular mechanisms in patients with RA remains lacking (45, 46).

Patients with RA may exhibit clinical features compatible with altered nociceptive processing, but available questionnaires do not by themselves establish nociplastic pain or central sensitization. In one painDETECT-based study, neuropathic-like pain features were identified in 22.5% of patients and were independently associated with pain intensity, tender joint count, and education level, but not with C-reactive protein (CRP), ESR, or the Disease Activity Score in 28 joints (DAS28) (5). Discordance between patient-reported and physician-assessed disease activity, particularly a high tender–swollen joint count difference and elevated subjective DAS28 components, may prompt assessment of non-inflammatory contributors to pain but is not diagnostic of central sensitization (47). Fibromyalgia is a clinical syndrome that may include nociplastic features but is not synonymous with nociplastic pain or central sensitization (41). It frequently coexists with RA, with pooled prevalence estimates of 18–24% (48). Patients with concomitant fibromyalgia have higher DAS28 and tender joint counts despite similar inflammatory markers, but these associations do not establish a single pain mechanism or direct CNS pathology (33, 49).

In a prospective observational study, inefficient conditioned pain modulation and non-nociceptive pain phenotypes were associated with poorer European Alliance of Associations for Rheumatology (EULAR) response and a lower likelihood of sustained DAS28 remission (50). Pain phenotyping may help avoid unnecessary escalation of immunosuppression when inflammation is controlled and guide adjunctive symptom-directed management (41). Clinical features and observational findings relevant to the proposed joint–brain axis are summarized in **Table 1**.

**Table 1:** Clinical features and observational findings relevant to the proposed joint–brain axis in rheumatoid arthritis.

Domain	Main features	Interpretation and evidence limitations	References
Persistent pain	Pain persisting despite low inflammatory activity or clinical remission in some cohorts	Not fully explained by routinely measured inflammatory activity; underlying mechanisms remain heterogeneous	(5, 16–18)
Nociplastic features	Widespread pain, hyperalgesia, allodynia, and fibromyalgia-related features	May indicate altered nociceptive processing but does not diagnose central sensitization or direct CNS pathology	(33, 47–49)
Cognitive symptoms	Difficulties in memory, attention, and executive function	Clinically important and multifactorial; direct RA-related CNS or glial mechanisms remain unconfirmed	(19–21)
Depression and anxiety	Mood symptoms associated with pain and impaired function	Associated with greater symptom burden and poorer functional outcomes; causal relationships may be bidirectional	(4, 6, 25, 51)
Fatigue	Persistent fatigue with variable or weak associations with inflammatory measures	Multifactorial; may involve pain, sleep, mood, inflammation, and potentially central processes	(26, 27, 35, 36)
Neuroimaging findings	Structural and functional brain differences reported in observational studies	Associations lack cellular specificity and require mechanistic validation	(12, 28, 29)

CNS, central nervous system; RA, rheumatoid arthritis.

## 3 Evidence for glial mechanisms in the proposed joint–brain axis

### 3.1 Routes of joint-derived and systemic inflammatory signaling to the CNS

#### 3.1.1 Humoral, cellular, neural, and neurovascular routes

Signals associated with inflamed joints and systemic RA inflammation may reach the CNS through humoral, cellular, neural, and neurovascular routes (52, 53). Evidence from broader neuroimmunology and experimental models indicates that circulating cytokines and chemokines can influence the brain through endothelial signaling, carrier-mediated transport, blood–brain barrier alterations, and circumventricular organs (52, 53). Human CSF observations are consistent with intrathecal cytokine signaling in RA but do not identify the precise tissue source, route of entry, or producing cell type (54). In experimental arthritis, cluster of differentiation 4-positive (CD4+) T-cell infiltration into the spinal dorsal horn was associated with glial activation and pain-like behavior, while collapsin response mediator protein 2 (CRMP2) inhibition attenuated these responses (45). The relative contributions of synovial inflammation and extra-articular systemic immune activation to the circulating signals reaching the CNS remain unresolved.

Neural signaling may involve joint-afferent, vagal, and autonomic pathways, while circumventricular organs may sense circulating inflammatory mediators. Experimental arthritis studies provide preclinical support for these routes (11, 55). Human vagus nerve stimulation studies provide evidence of therapeutic neuroimmune modulation but do not establish the physiological routes of joint-to-brain signaling in RA (13, 56); detailed clinical evidence is discussed in Section 4.1.1.

Taken together, the available evidence supports several plausible neural, humoral, cellular, neuroendocrine, and neurovascular routes through which joint-derived neural inputs and systemic inflammatory signals associated with RA may influence CNS glial and glio-vascular responses; most pathway-level evidence remains preclinical (**Figure 1A**).

#### 3.1.2 Temporal and spatial dynamics of glial responses

Preclinical arthritis models suggest that spinal microglial responses emerge early, sometimes before visible joint swelling, whereas astrocytic responses tend to appear later and may contribute to persistent hypersensitivity (15, 57). Pharmacological silencing of joint afferents prevented early spinal microglial activation and allodynia, linking peripheral nociceptive input to the initial central response (58). Vascular and immune-cell mechanisms may contribute to subsequent glial recruitment (59).

Experimental arthritis studies have reported supraspinal glial responses in the hippocampus and area postrema (11, 28). Human evidence remains indirect: hippocampal metabolite abnormalities detected by proton magnetic resonance spectroscopy in juvenile idiopathic arthritis do not demonstrate glial activation and cannot be considered direct evidence in adult RA (60).

In a collagen-induced arthritis mouse model, area postrema microglial activation persisted into chronic disease phases, while a separate study in collagen-induced arthritis rats identified blood–brain barrier dysfunction and altered amyloid-β transport (11, 61). Whether these abnormalities persist after peripheral inflammation resolves has not been established in experimental arthritis or patients with RA.

### 3.2 Molecular mechanisms of glial activation

#### 3.2.1 Spinal level

At the spinal level, experimental arthritis and related joint-pain models have identified convergent microglial and astrocytic pathways associated with central sensitization and mechanical hypersensitivity (15). In a rodent model of joint pain, adenosine triphosphate (ATP) released from primary afferent terminals activated pannexin-1 and P2X purinoceptor 7 (P2X7) signaling in spinal microglia, promoting IL-1β release and mechanical allodynia (62). In collagen-induced arthritis, intrathecal P2X7 blockade attenuated allodynia, spinal microgliosis, and cerebrospinal fluid IL-1β before overt joint swelling (63). Downstream activation of the p65 subunit of nuclear factor kappa B (NF-κB), the NOD-like receptor family pyrin domain-containing 3 (NLRP3) inflammasome, and p38 mitogen-activated protein kinase (p38 MAPK) further promotes pro-inflammatory cytokine and BDNF production (64).

Evidence for signaling involving cathepsin S and C-X3-C motif chemokine receptor 1 (CX3CR1) is derived from preclinical pain models, including monoarthritis and non-RA visceral hypersensitivity models (15, 65). In monoarthritic rodents, CX3CR1 blockade delayed or reversed mechanical allodynia and reduced spinal microglial activation (15). In rats with collagen-induced arthritis, spinal NR2B phosphorylation and ERK2 activation were associated with mechanical hypersensitivity, while N-methyl-D-aspartate (NMDA) receptor antagonism attenuated pain-like behavior (46).

In experimental arthritis, CD4+ T cells infiltrated the spinal dorsal horn and contributed to glial activation and pain-like behavior; CRMP2 inhibition or conditional deletion reduced T-cell migration, glial activation, and hypersensitivity (45). In monoarthritic rats, bidirectional signaling between infiltrating T helper 1 (Th1) cells and astrocytes through interferon-γ (IFN-γ) was associated with persistent allodynia (66). Together, these preclinical findings identify several candidate pathways contributing to chronic pain-like behavior in experimental arthritis (15).

#### 3.2.2 Brain level

IL-1β is a candidate mediator of supraspinal dysfunction. In the human arm of a translational study, patients with RA had higher CSF IL-1β concentrations than controls, although CSF analysis did not identify the cellular source (54). In the accompanying K/BxN serum-transfer model, spinal IL-1β, IL-18, and TNF expression increased with arthritis severity (54). In collagen-induced arthritis mice, IL-1β-positive microglia accumulated in the area postrema and were associated with anhedonia-like behavior (11). In rat organotypic hippocampal cultures unrelated to arthritis, IL-1β impaired BDNF-dependent synaptic plasticity through p38 MAPK signaling (67).

In mice with collagen-induced arthritis, hippocampal enrichment of ionized calcium-binding adaptor molecule 1 (IBA1)-positive microglia and altered insulin-like growth factor 1 receptor (IGF1R)–insulin receptor substrate 1 (IRS1) signaling were associated with reduced neurogenesis, smaller hippocampal volume, and depression-like behavior; pharmacological inhibition of IGF1R partially restored neurogenesis (28). The corresponding human imaging arm did not assess the cell-specific IGF1R/IRS1 pathway (28).

In adjuvant-induced arthritis rats, neuronal and endothelial BDNF–tropomyosin receptor kinase B (TrkB) signaling and endothelial nitric oxide synthase activity were reduced; arginase inhibition attenuated brain BDNF deficiency without improving peripheral inflammation (68, 69). In patients with RA, lower circulating BDNF was associated with depression severity, but circulating levels do not establish CNS pathway activity (25). By contrast, signaling involving precursor brain-derived neurotrophic factor (proBDNF) and the p75 neurotrophin receptor (p75NTR) was implicated in proinflammatory responses in collagen-induced arthritis mice and human RA synovial tissue, representing a distinct, predominantly peripheral pathway whose relevance to CNS-related symptoms remains uncertain (70). Thus, the apparently divergent effects of BDNF are likely context dependent and may vary according to molecular form, receptor, cellular source, and anatomical compartment. Mature BDNF/TrkB signaling may support neuronal and neurovascular plasticity in supraspinal regions, whereas microglia-derived BDNF within spinal nociceptive circuits and proBDNF/p75NTR signaling may contribute to pronociceptive or proinflammatory responses (64, 67–70).

#### 3.2.3 Glio-vascular unit

In cultured brain microvascular endothelial cells stimulated with vascular endothelial growth factor A (VEGF-A) and TNF-α, vascular endothelial growth factor receptor 2 (VEGFR2) inhibition reduced intercellular adhesion molecule 1 (ICAM-1)-dependent monocyte adhesion (59); a separate endothelial-cell study linked VEGF signaling to protein kinase D-dependent production of IL-6, IL-8, and growth-regulated oncogene-α (GRO-α) (71). In intra-articular complete Freund’s adjuvant-induced arthritis, pharmacological VEGFR2 inhibition in rats and endothelial-specific VEGFR2 deletion in mice attenuated spinal endothelial activation, perivascular microglial accumulation, and mechanical allodynia (59).

Inflammasome-dependent signaling is supported mainly by cultured glial-cell studies and non-RA neuroinflammatory models, in which lipid-raft organization influences microglial IFN-γ receptor trafficking and inflammasome-dependent IL-1β release (72–75). Reviews of broader non-RA models suggest that reactive astrocyte-derived VEGF-A and proinflammatory cytokines may disrupt endothelial junctions, but these mechanisms have not been demonstrated in experimental arthritis or patients with RA (76, 77).

### 3.3 Symptom-specific interpretation of glial evidence

#### 3.3.1 Chronic pain and central sensitization

Preclinical arthritis models support spinal glial activation as a potential contributor to persistent pain-like behavior. Interventions that reduced joint-afferent input or immune-cell recruitment attenuated glial responses and mechanical hypersensitivity (45, 58), while related studies implicated high-mobility group box 1 (HMGB1) and spinal cytokine signaling (15, 78, 79). Together, these preclinical findings provide a mechanistic basis for investigating persistent pain in patients with RA despite controlled synovitis.

#### 3.3.2 Cognitive symptoms and neurobiological changes

Preclinical arthritis studies associate hippocampal and neurovascular abnormalities with cognitive and behavioral changes, including impaired neurogenesis, blood–brain barrier dysfunction, glutamatergic dysregulation, and memory deficits (28, 61, 80). Human neuroimaging studies have also reported associations between RA and brain alterations, while cognitive symptoms may reflect pain, mood disturbance, fatigue, vascular comorbidity, and treatment effects (2, 28). Together, these findings support consideration of candidate glial-related mechanisms within a broader, multifactorial framework of cognitive symptoms in RA.

#### 3.3.3 Fatigue and neuropsychiatric symptoms

Human CSF observations and preclinical area postrema findings suggest that IL-1β-related pathways may be relevant to fatigue and affective symptoms (11, 54). Monoaminergic, kynurenine, fractalkine–CX3CR1, NMDA receptor, and BDNF pathways provide additional hypotheses derived mainly from mechanistic reviews and preclinical studies (51, 81). Fatigue and neuropsychiatric symptoms may reflect interactions among these candidate pathways, pain, sleep disturbance, systemic inflammation, treatment effects, and psychosocial factors.

## 4 Bidirectional communication: evidence for CNS modulation of peripheral arthritis

Potential central-to-peripheral influences in RA include vagal/autonomic, neuroendocrine, spinal neuroimmune, and behavioral pathways that may influence peripheral inflammatory activity, symptom burden, or both. Evidence supporting these pathways derives from rodent intervention studies, human observational studies, and clinical neuromodulation trials, with substantial differences in mechanistic specificity and causal strength (**Figure 1B**).

### 4.1 Descending anti-inflammatory pathways

#### 4.1.1 Vagal anti-inflammatory reflex

The cholinergic anti-inflammatory pathway has been defined largely through preclinical mechanistic studies. In experimental arthritis, vagus nerve stimulation or α7 nicotinic acetylcholine receptor (α7nAChR) activation reduced cytokine production, joint destruction, and clinical arthritis severity (82). In an 84-day open-label proof-of-concept study of patients with RA, implantable vagus nerve stimulation reduced ex vivo TNF production and was accompanied by improved disease activity (56). In the subsequent 242-patient, double-blind, sham-controlled RESET-RA trial of implantable vagus nerve stimulation in patients with moderate-to-severe RA, active stimulation produced a higher American College of Rheumatology 20% (ACR20) response rate than sham stimulation at 3 months (13). After 3 months, both groups received active stimulation; therefore, the reported 12-month improvements represent open-label follow-up rather than a sustained sham-controlled comparison. A small uncontrolled pilot study further suggested that non-invasive cervical vagus nerve stimulation may reduce DAS28-CRP, CRP, and IFN-γ, but these findings require controlled confirmation (83).

#### 4.1.2 Neuroendocrine regulation

Neuroendocrine regulation is supported by a combination of physiological, clinical observational, and preclinical evidence. Clinical studies and reviews have reported relative hypothalamic–pituitary–adrenal axis hypofunction in some patients with RA, although the direction and clinical importance of this association remain uncertain (84). In mouse arthritis models, glucocorticoid receptor signaling in stromal cells contributed to the anti-inflammatory effects of glucocorticoids (85). Experimental studies also indicate that β2-adrenergic signaling can inhibit T helper 1 and T helper 17 responses, while observational tissue studies have documented altered sympathetic innervation and adrenergic signaling in RA synovium (86, 87). A recent experimental arthritis study implicated hypothalamic tyrosine hydroxylase-positive neurons in a neuroimmune feedback loop that increased peripheral inflammation; direct evidence for this pathway in patients with RA is currently unavailable (88).

### 4.2 Preclinical evidence from spinal cytokine modulation

#### 4.2.1 Evidence from intrathecal interventions

All evidence in this subsection comes from rat arthritis models. In rats with inflammatory knee arthritis, spinal etanercept or intrathecal p38 inhibition reduced spinal hyperexcitability and peripheral arthritis severity (89). In rats with adjuvant-induced arthritis, intrathecal knockdown targeting spinal expression of either IL-33 or the p65 subunit of nuclear factor kappa B (NF-κB) attenuated hyperalgesia, joint swelling, and structural damage (90, 91). In a separate rat knee-inflammation model, spinal IL-6 trans-signaling blockade limited the development, but not the maintenance, of hyperexcitability (92). Together, these studies suggest that spinal cytokine pathways can modulate pain processing and peripheral arthritis in rats; equivalent CNS-directed interventions have not been tested in patients with RA.

#### 4.2.2 Translational implications for centrally acting therapies

The rat intrathecal studies reviewed above provide a rationale for testing whether CNS-directed interventions can influence both nociceptive processing and peripheral arthritis, but such dual effects have not been demonstrated in patients with RA (89–92). Human neuroimaging reports have described treatment-associated changes in pain-related brain activation after TNF inhibition (37, 38), but these observations do not distinguish direct CNS effects from reduced systemic inflammation, altered nociceptive input, or changes in symptom perception. Direct effects of IL-6 or Janus kinase (JAK) inhibitors on central pain pathways or glial activity in patients with RA remain unestablished (93, 94).

### 4.3 Behavioral and psychological factors

#### 4.3.1 Stress-induced exacerbation

Across a prospective diary study, a cross-sectional pandemic survey, and an 18-month longitudinal cohort, daily stressors, worrying, perceived or financial stress, and poor sleep were associated with greater reported disease activity, flares, pain, fatigue, and functional impairment (95–97). Preclinical studies further suggest that chronic stress may aggravate experimental arthritis through cytokine, neuroendocrine, oxidative, and immune pathways (98). In a standardized psychosocial stress-test study, patients with RA showed greater stress-induced IL-1β and IL-2 responses than patients with psoriasis and healthy controls, although the clinical relevance of these cytokine responses remains uncertain (99).

#### 4.3.2 Mind-body interventions

Mind–body interventions have been evaluated as adjunctive approaches for symptom management in RA, although evidence for direct modulation of joint–brain pathways remains limited. Randomized trials in patients with RA have reported improvements in pain interference, depression, mood, anxiety, and selected functional or psychological outcomes after online mindfulness or cognitive behavioral therapy, with some benefits persisting for 12 months (100, 101). Meta-analyses across heterogeneous clinical populations have reported variable changes in CRP, IL-6, and other immune markers after psychological interventions, but these findings are not specific to RA (102, 103). In an 8-week randomized trial of patients with RA, a yoga-based lifestyle intervention was associated with improved disease activity and quality of life and with changes in inflammatory and neuroendocrine biomarkers (104).

## 5 Therapeutic implications: current treatments and investigational strategies

Current RA therapies primarily target peripheral inflammation, whereas persistent pain and other multifactorial symptoms may require additional assessment and management. This section evaluates CNS-related outcomes associated with disease-modifying antirheumatic drugs (DMARDs) and the evidence for adjunctive therapeutic approaches. **Figure 2** summarizes a multidimensional framework integrating clinical assessment, persistent symptom burden, current management, and future translational directions in rheumatoid arthritis.

**Figure 2:**
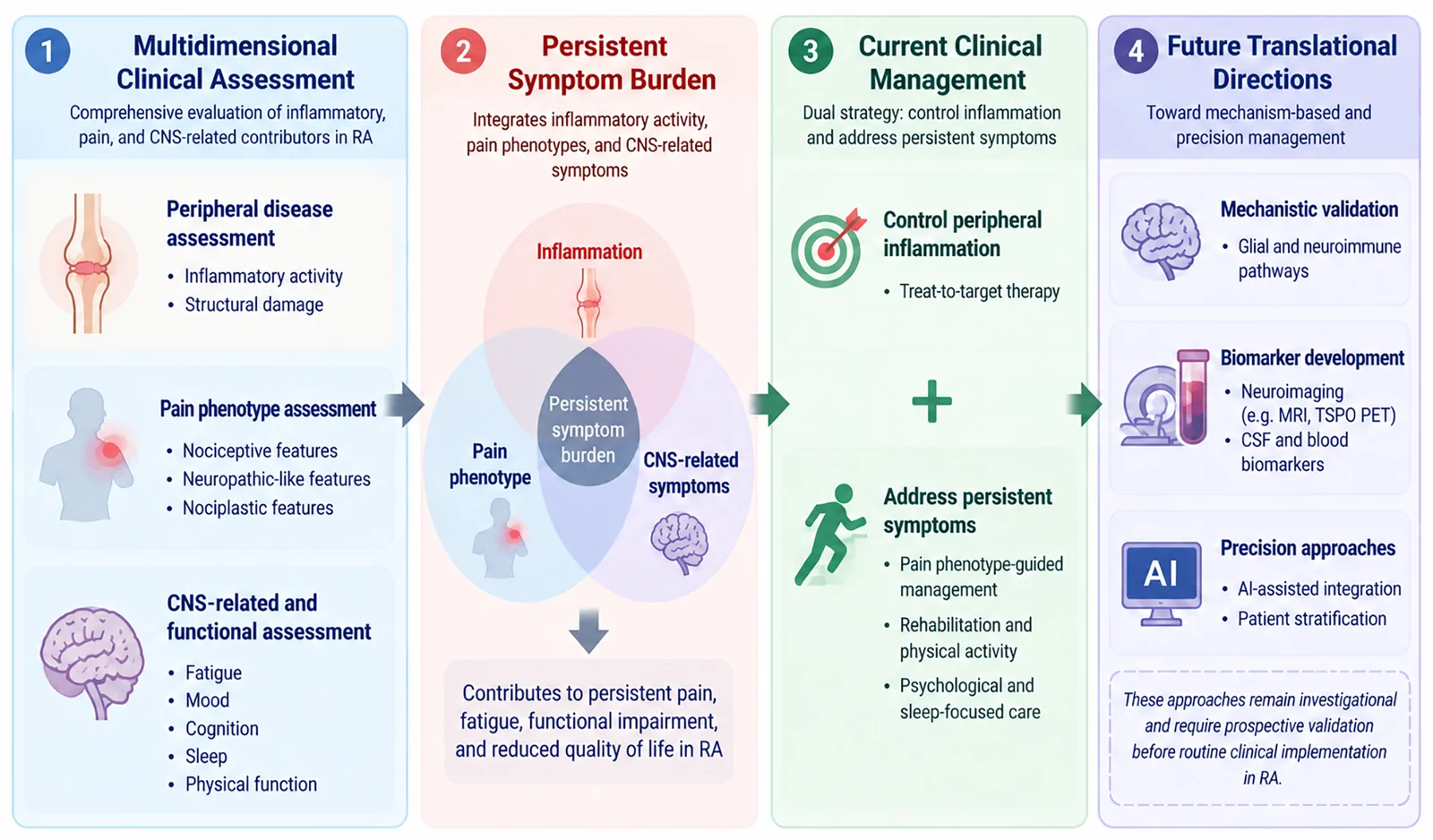
Clinical assessment and translational research framework for persistent symptom burden in rheumatoid arthritis. Multidimensional clinical assessment integrates evaluation of peripheral inflammatory activity and structural damage, pain phenotypes (nociceptive, neuropathic-like, and nociplastic features), and central nervous system (CNS)-related and functional symptoms, including fatigue, mood, cognition, sleep, and physical function. Persistent symptom burden represents the integrated impact of inflammatory activity, pain phenotypes, and CNS-related symptoms, contributing to persistent pain, fatigue, functional impairment, and reduced quality of life. Current management adopts a dual strategy combining treat-to-target control of peripheral inflammation with phenotype-guided management of persistent symptoms, including rehabilitation, physical activity, and psychological and sleep-focused interventions. Future translational directions, including mechanistic validation, biomarker development, and precision approaches, remain investigational and require prospective validation before routine clinical implementation.

RA, rheumatoid arthritis; CNS, central nervous system; MRI, magnetic resonance imaging; TSPO PET, translocator protein positron emission tomography; CSF, cerebrospinal fluid; AI, artificial intelligence.

### 5.1 CNS-related outcomes associated with current DMARDs

#### 5.1.1 TNF inhibitors

Human evidence for CNS-related outcomes associated with TNF inhibitors includes prospective cognitive assessment, small neuroimaging studies, and observational cohorts. In a prospective study of 70 patients with RA, cognitive scores improved after 6 months of TNF inhibitor treatment (105). In the 18-month RESIST observational study of patients with RA and mild cognitive impairment, cognitive scores remained stable in both the TNF inhibitor and conventional synthetic DMARD groups, with no significant between-group differences (106). Functional MRI studies reported treatment-associated changes in pain-related brain activation and connectivity (38, 39), while the randomized, multicenter, double-blind, placebo-controlled phase 3 PreCePra trial in patients with RA evaluated disease-associated brain activation as a predictor of clinical response to TNF inhibition (107). Retrospective cohorts, database studies, and meta-analyses have reported associations between TNF inhibitor use and lower dementia incidence (108–110). These findings remain susceptible to treatment-selection bias and confounding by systemic inflammation control and do not establish direct CNS or glial target engagement or neuroprotection.

#### 5.1.2 IL-6 inhibitors and JAK inhibitors

As reviewed in Section 4.2, evidence from a rat model indicates that spinal IL-6 signaling contributes to inflammation-induced hyperexcitability, whereas corresponding CNS effects of JAK inhibition remain hypothetical. In a national claims analysis, biological or targeted synthetic DMARD use was associated with lower dementia incidence than conventional synthetic DMARD treatment, but risk reductions were comparable across drug classes (111). A VigiBase disproportionality analysis identified disproportionate reporting of memory impairment with JAK inhibitors, although these data cannot establish incidence or causality (112). Overall, evidence regarding CNS or glial effects of IL-6 and JAK inhibitors in patients with RA remains limited.

### 5.2 Adjunctive and investigational strategies

#### 5.2.1 Candidate glial modulators

In the 48-week randomized, double-blind, placebo-controlled Minocycline in Rheumatoid Arthritis (MIRA) trial involving patients with RA, minocycline improved peripheral disease measures, but CNS or glial outcomes were not assessed (113, 114). In rat neuropathic pain models, ibudilast reduced established allodynia and phosphorylated p38-positive spinal cells, whereas minocycline produced different effects on microglial phenotypes (115). Ibudilast also suppressed chemokine expression in cultured human RA synovial fibroblasts and reduced disease progression in collagen-induced arthritis mice, without assessment of CNS outcomes (116). A systematic review across chronic pain conditions found inconsistent clinical efficacy, and no trial has directly evaluated glial target engagement in patients with RA (117).

#### 5.2.2 Pathway-specific targets

Rodent experimental arthritis studies identify CRMP2 and endothelial VEGFR2 as candidate targets: inhibition or conditional deletion of these pathways attenuated spinal immune-cell or glio-vascular responses and pain-like behavior (45, 59). The P2X7–cathepsin S–fractalkine/CX3CR1 pathway is supported by broader preclinical pain and arthritis studies (64, 65, 92). None of these pathways has demonstrated CNS target engagement, efficacy, or safety in patients with RA; human translation will require pathway-specific evidence beyond changes in peripheral inflammation.

#### 5.2.3 Centrally acting pain medications

Evidence for centrally acting analgesics in RA is largely extrapolated from other chronic pain conditions. Meta-analyses across heterogeneous chronic pain populations support modest analgesic effects of serotonin–norepinephrine reuptake inhibitors, particularly duloxetine, but dedicated RA trials remain limited (118–121). Clinical evidence for gabapentinoids in inflammatory pain is inconsistent, and efficacy in patients with RA has not been established (122, 123). In experimental arthritis, gabapentin attenuated peripheral nerve sensitization through NR2B-, transient receptor potential vanilloid 1 (TRPV1)-, and cytokine-related mechanisms (124), but this preclinical finding does not establish efficacy against nociplastic pain in RA. These agents should therefore be considered adjunctive and evaluated according to pain phenotype, comorbidities, and safety considerations.

### 5.3 Emerging biomarker and patient-stratification approaches

#### 5.3.1 Neuroimaging as a research tool

Available evidence for MRI-based stratification in RA is limited to human observational studies. In a UK Biobank analysis, reduced white matter fractional anisotropy statistically mediated associations of RA with depression, sleep disturbance, and dementia-related outcomes (31), although the clinical significance of these associations requires prospective validation. Separate observational studies associated baseline joint MRI features with subsequent treatment response or structural progression (125, 126); these findings concern peripheral disease monitoring rather than CNS stratification. No prospective imaging-guided interventional study has validated brain MRI for selecting CNS-directed treatment in patients with RA.

#### 5.3.2 CSF biomarker candidates

Human evidence for CSF-based stratification in RA is limited to small exploratory studies. The available IL-1β study did not establish the cellular source or diagnostic and predictive performance of CSF IL-1β (54). In a paired proteomic analysis of seven patients with inflammatory polyarthritis, TNF blockade was associated with reductions in 35 inflammation-related CSF proteins, several of which correlated with systemic inflammation, disability, or pain improvement (127). These studies remain exploratory, and no CSF biomarker has been prospectively validated for pain phenotyping or treatment selection in RA.

#### 5.3.3 Proposed patient-stratification frameworks

Research-oriented stratification should integrate peripheral inflammatory activity, pain phenotype, patient-reported outcomes, and quantitative pain assessment. Brain MRI and CSF measures may be included as exploratory variables, but current support is limited to observational imaging studies and small CSF analyses and does not establish clinical utility for treatment selection (31, 125–127). Prospective studies should determine whether these measures provide incremental value beyond routine clinical assessment before their incorporation into practice. Current clinical and investigational approaches relevant to the proposed joint–brain axis are summarized in **Table 2**.

**Table 2:** Current clinical and investigational approaches relevant to the proposed joint–brain axis in rheumatoid arthritis.

Approach	Potential use	Main limitation	References
Pain phenotyping	Supports characterization of nociceptive, neuropathic-like, and nociplastic features	No questionnaire alone confirms nociplastic pain, central sensitization, or direct CNS pathology	(5, 40, 42, 47, 50, 93)
Patient-reported outcomes	Captures pain, fatigue, mood, sleep, function, and treatment-associated symptom changes	Subjective, multifactorial, and context-dependent; does not provide direct evidence of CNS or glial involvement	(6, 26, 27, 93)
Brain MRI	Provides exploratory characterization of structural and functional brain associations	Findings are observational and nonspecific; protocols are heterogeneous, and treatment-selection utility remains unvalidated	(12, 28–31, 35, 39)
CSF biomarkers	Provides exploratory assessment of intrathecal inflammatory signals	Invasive and generally cell-nonspecific; no marker has been validated for clinical stratification or treatment selection	(54, 127)
TNF inhibitors	Human studies have evaluated cognitive and neuroimaging outcomes during treatment	Findings are mixed and susceptible to confounding; direct CNS or glial effects remain unestablished	(38, 39, 105–110)
IL-6/JAK inhibitors	Provide established peripheral inflammation control; CNS-related effects remain investigational	Direct human CNS or glial target engagement has not been demonstrated; available evidence is mainly observational or pharmacovigilance-based	(94, 111, 112, 128)
Behavioral interventions	May improve pain interference, mood, fatigue, coping, or other patient-reported outcomes	Interventions and outcomes are heterogeneous, and reported biomarker effects are not specific to RA	(100–104)
Neuromodulation	Investigationally targets autonomic–immune signaling, with clinical responses reported in selected RA studies	Mechanisms, durability, patient selection, and broader clinical effectiveness require further validation	(13, 56, 83)

CNS, central nervous system; CSF, cerebrospinal fluid; IL-6, interleukin-6; JAK, Janus kinase; MRI, magnetic resonance imaging; RA, rheumatoid arthritis; TNF, tumor necrosis factor.

## 6 Evidence gaps and future directions

### 6.1 Limitations of the current evidence

Direct evidence of cell-specific CNS glial activation in patients with RA is scarce. Most pathway-level evidence derives from rodent arthritis models, cultured cells, or non-RA inflammatory pain models, and heterogeneity in species, model, disease stage, sex, and outcome measurement limits extrapolation to human RA (11, 15, 45, 59).

Human evidence is dominated by small observational cohorts, patient-reported outcomes, and neuroimaging or CSF measures with limited cellular specificity. CNS-related symptoms are multifactorial, and their associations with inflammatory activity or treatment-associated changes provide limited information regarding cellular mechanisms and CNS target engagement. The relative contributions of joint-derived and systemic inflammatory signals also remain unresolved. Accordingly, the proposed joint–brain axis remains a provisional framework, and neuroimaging and CSF measures remain research tools.

### 6.2 Unresolved mechanistic questions and human validation

Key unresolved questions are whether CNS glial responses occur in patients with RA, which cell states and regions are involved, and whether these responses persist independently of peripheral inflammation. Single-cell and spatial analyses may characterize glial and vascular cell states in available tissue, while 18-kDa translocator protein positron emission tomography (TSPO PET) and macrophage-targeted imaging may support longitudinal *in vivo* assessment (129–132). However, tissue access is limited, imaging signals lack cell specificity, and none of these approaches has been validated in well-characterized RA cohorts. Longitudinal human studies should relate these measures to inflammatory activity and clinical phenotypes before defining the clinical relevance of glial-related mechanisms.

### 6.3 Clinical trial design and translational readiness

Future trials should enroll patients using predefined inflammatory activity and pain phenotypes and test whether interventions directed at central pain processing provide additional benefit when synovitis is adequately controlled (93). Neuroimaging, blood-based markers, and selected CSF measures may be included as exploratory endpoints, with prespecified assessment of their incremental value beyond clinical evaluation (31, 127, 133). Longer-term studies in clinically defined high-risk cohorts may assess whether early intervention reduces persistent pain or cognitive and neuropsychiatric decline; glial activation should be used as an endpoint only when a sufficiently specific and validated measure is available (32).

### 6.4 Practical implications for multidimensional clinical assessment and management

Routine assessment should document pain intensity with a numeric rating scale or visual analog scale and interpret it alongside evidence of active synovitis or structural joint damage. painDETECT may screen for neuropathic-like features, the Central Sensitization Inventory may capture symptoms associated with central sensitivity syndromes, and the Widespread Pain Index and Symptom Severity Scale may support evaluation for concomitant fibromyalgia (5, 40, 93). None of these questionnaires alone confirms nociplastic pain or central sensitization. Discordance between inflammation and pain should prompt assessment of pain distribution, sleep, fatigue, mood, comorbidities, medication effects, and function (47, 93). Cognitive complaints may warrant brief MoCA screening and specialist referral (134). This multidimensional assessment may guide adjunctive symptom-directed management. Brain MRI, CSF measures, and candidate glial biomarkers remain research tools for joint–brain-axis phenotyping and treatment selection pending prospective validation.

## 7 Conclusions

Patients with RA experience a substantial burden of persistent pain, fatigue, cognitive symptoms, and mood disturbance. These multifactorial manifestations highlight the importance of considering peripheral inflammation alongside altered pain processing and broader clinical and psychosocial factors. The proposed joint–brain axis provides a framework for integrating human neuroimaging, CSF, symptom, and neuromodulation findings with glial mechanisms identified predominantly in preclinical models. Clinical assessment should combine inflammatory activity with pain phenotype and broader symptom burden to guide complementary symptom-directed management. Future studies should evaluate the incremental value of neuroimaging and CSF measures for patient stratification and test whether glial- or pathway-directed strategies improve outcomes in well-defined clinical populations.
